# Foreign Body Migration from Subglottis to Bronchus in a Tracheostomised Child: A Case Report

**DOI:** 10.31729/jnma.9141

**Published:** 2025-07-31

**Authors:** Ashok Aryal, Leison Maharjan, Deepak Yadav

**Affiliations:** 1 Department of Otolaryngology and Head & Neck Sugery, United Mission Hospital, Palpa, Nepal; 2 Department of Otolaryngology and Head & Neck Sugery, Patan Academy of Health Sciences, Lalitpur, Nepal.

**Keywords:** *foreign body*, *foreign body migration*, *rigid bronchoscopy*, *tracheostomy*

## Abstract

Foreign body (FB) aspiration is a critical paediatric emergency, often requiring timely diagnosis and intervention to prevent complications. We report the case of a three-year-old female presenting with persistent cough and breathing difficulty, initially managed as croup. Imaging revealed a subglottic FB, necessitating tracheostomy for airway stabilization. During the first rigid bronchoscopy attempt, the FB migrated into the left main bronchus, likely due to manipulation and the use of an uncuffed tracheostomy tube. A second bronchoscopy successfully removed the FB, a 2.5*2 cm bone chip, using telescope-mounted forceps. This case highlights the rare phenomenon of FB migration during intervention and emphasizes the importance of tracheostomy in maintaining airway. Awareness of migration risks and tailored interventions are critical for successful outcomes.

## INTRODUCTION

Aspiration of a foreign body (FB) in children causes 350-2,000 annual deaths in the United States.^1,2^ Children below three years of age are at higher risk due to poor pharyngeal reflexes, incomplete dentition, and their exploratory habits.^1^ Foreign bodies generally lodge in the bronchial trees on right middle and lower bronchi.^3,4^ Delay in diagnosis and treatment can lead to serious complications in up to 6% of cases.^5,6^ Rigid bronchoscopy under general anesthesia is an effective management for FB removal.^7^ Tracheostomy is required in 0.3-4% of cases as an open operative method.^8^

## CASE REPORT

A 3-year-old female presented with two days history of cough and difficulty in breathing. She was initially admitted in pediatric ward with a diagnosis of croup and treated with oxygen and steroids. Lack of symptomatic improvement on the following day raised suspicion of FB. Further questioning of the parents revealed a history of sudden onset of coughing while eating. She was initially treated at a local health center with nebulization but was referred to a higher center after failing to improve.

**Figure 1:**
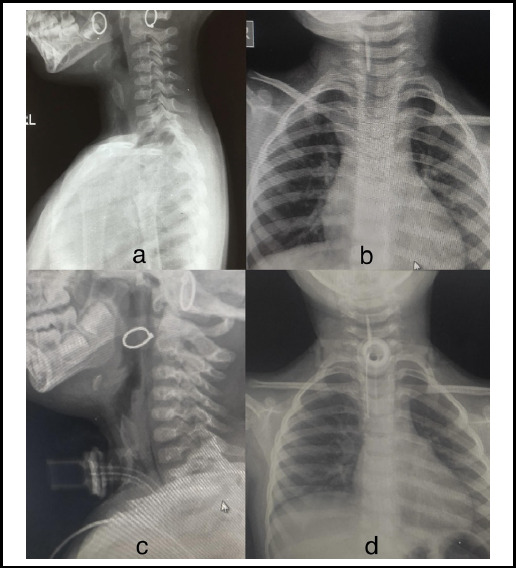
X-ray showing Subglottic FB: Before tracheostomy a) Antero-posterior b) Lateral view and After tracheostomy c) Antero-posterior d) Lateral view.

At our center, United Mission Hospital, X-ray soft tissue neck revealed a FB lodged in the subglottic airway (Figure 1).

**Figure 2:**
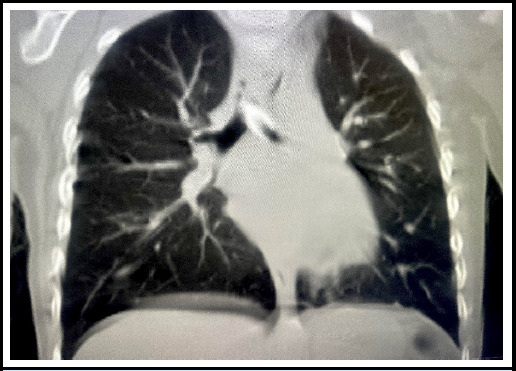
Computed Tomography (CT) scan of Chest revealing foreign body in left main bronchus.

The family members were counseled regarding the need rigid bronchoscopy. Due to the unavailability of rigid bronchoscope at our center, family was counselled about the need of tracheostomy for safe transfer. Then, the patient was referred to Patan Academy of Health Sciences (PAHS) with an uncuffed tracheostomy tube. At PAHS, rigid bronchoscopy was attempted; however, the FB was not visualised upon advancing the bronchoscope into the trachea. Flexible bronchoscopy through the tracheostomy tube also failed to locate the FB up to the primary bronchus. Due to the child’s unstable condition, the tracheostomy tube was left in place, and the procedure was abandoned. A post-operative CT scan revealed the FB had migrated to the left main bronchus (Figure 2).

**Figure 3:**
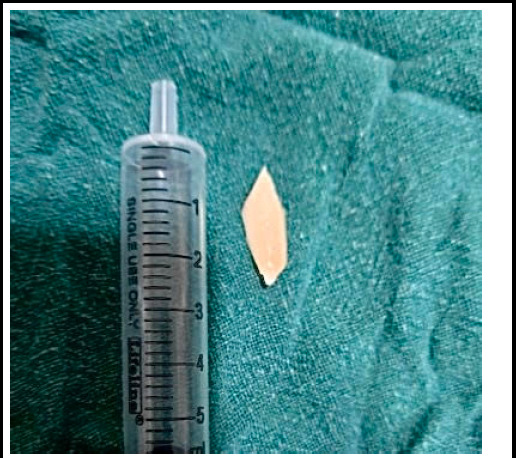
Foreign body bone chip of 2.5 × 2 cm.

An initial rigid bronchoscopy was performed without removing the tracheostomy tube, as the foreign body (FB) was located above its level. During attempted extraction, the FB became dislodged. Subsequent flexible bronchoscopy through the tracheostomy tube failed to visualize the FB up to the primary bronchi, and the procedure was aborted due to hemodynamic instability. A second rigid bronchoscopy was performed 8 to 10 hours later following removal of the tracheostomy tube. The FB was advanced to the subglottic region, the tracheostomy tube was reinserted, and the FB was successfully extracted using telescope-mounted forceps. A bone chip measuring 2.5*2 cm was retrieved (Figure 3).

The prolonged surgery and mucosal inflammation and tears necessitated keeping the child in high care with the tracheostomy tube in place. Intravenous antibiotics and steroids are prescribed to reduce the airway inflammation. On the fourth postoperative day, decannulation was performed successfully, patient tolerated without tracheostomy tube and was discharged on the same day.

## DISCUSSION

Foreign body aspiration is a common and underestimated problem in children, but migration of the FB is a rare occurrence.^1,6^ FB aspiration of the airway is preventable cause of morbidity and mortality among the both paediatric and adult population.^1^ Most sites of migration are bronchus, lungs, or esophagus.^2,6,9^ Clinical presentation depends on the site of impaction, shape of the foreign body, nature and degree of obstruction^12^; however, the triad of cough, wheeze, and decreased breath sounds is common.^5^ Asphyxia can occur in case of complete obstruction of the airway leading to death.^12^ In children, round, ovoid and flexible objects causes more obstruction which often is partial.^12^ Migration is facilitated by increased expiratory flow rates, which can reach up to 12 L/s during coughing. Inorganic and smooth objects are more prone for migration.^2,6,10^ In this case, the FB migrated from the larynx to the main bronchus. The nature and shape of the FB significantly influence the likelihood of migration.

In our case, detail history of the patient gave clue for foreign body and respiratory symptoms explain the airway problem. Plain radiography of the neck in both view (antero-posterior and lateral) is the ideal and initial diagnostic tool for assessment and localization of FB in the airway.^5^ The both view helps to distinguish from airway and aerodigestive tract. Here the FB was initially lodged just below the glottic area which later migrated to lower airway tract. Right bronchial tree is the common site of FB lodgement (50%) followed by left bronchus in 44%.^1^ Our case study also shows the migration to the left bronchus.

FBs in the larynx are less common than those in the bronchus but pose greater clinical challenges due to increased risk of airway compromise and difficult management.^8^ Sharp or pointed objects rarely migrate once lodged in the mucosa. Organic materials can cause severe inflammatory responses, absorb water, and exacerbate airway obstruction.^11^ However, inorganic materials are inert in nature and sometimes may be tolerated for many years without prominent symptoms.^2^ In our case also being organic tissue cause inflammatory response and had clinical symptoms. But the suspected bone did not swell up so easily migrated from the initial location. It is hypothesized that during the initial rigid bronchoscopy, the FB may have been pushed further down the airway and subsequently migrated to the bronchus. In this case, the FB’s thin nature and potential gap between the uncuffed tube and tracheal wall. Dislodgement during manipulation might explain its migration.

Rigid bronchoscopic is the ideal method for FB removal from the airway.^8^ In our case we initially tried with rigid bronchoscope even after identification of FB location after imaging (CT scan). Endoscopic removal of the FBs sometime in experienced endoscopist also need to convert into open procedure like tracheostomy, thoracotomy or even bronchotomy and lung resection. Lodgment of the FB in distal airway tract is one of the indications for tracheostomy. However, tracheostomy in the laryngotracheal FBs make the extraction process easy, safe without tearing the vocal cords and subglottic area.^8^ In our case, we performed tracheostomy to secure the airway during transfer to PAHS.

## CONCLUSION

Regardless of the nature and type of FB, physicians must remain vigilant about the possibility of migration of FB within the airway, including the trachea and bronchus. Rigid bronchoscopy is the mainstay of FB retrieval from the airway. Tracheostomy is done to secure the airway before attempting FB removal depending on the clinical feature and expertise of surgeon. In places where rigid bronchoscopy is not available, tracheostomy helps to secure the airway during their transfer to the tertiary center.
